# Traditional Chinese Medicine Treatment Associated with Female Infertility in Taiwan: A Population-Based Case-Control Study

**DOI:** 10.1155/2020/3951741

**Published:** 2020-12-08

**Authors:** Yueh-Hsiang Liao, Jaung-Geng Lin, Cheng-Chieh Lin, Chin-Chuan Tsai, Hui-Lien Lai, Tsai-Chung Li

**Affiliations:** ^1^School of Chinese Medicine for Post-Baccalaureate, I-Shou University, Kaohsiung, Taiwan; ^2^School of Chinese Medicine, College of Chinese Medicine, China Medical University, Taichung, Taiwan; ^3^School of Medicine, College of Medicine, China Medical University, Taichung, Taiwan; ^4^Department of Family Medicine, China Medical University Hospital, Taichung, Taiwan; ^5^Nantou Hospital, Ministry of Health and Welfare, Taipei, Taiwan; ^6^Department of Public Health, College of Public Health, China Medical University, Taichung, Taiwan; ^7^Department of Healthcare Administration, College of Medical and Health Science, Asia University, Taichung, Taiwan

## Abstract

**Background:**

Traditional Chinese medicine (TCM) for the treatment of female infertility remains ambiguous. The aim of the present case-control study was to examine the association between TCM treatment and successful pregnancy among infertile women.

**Methods:**

This population-based case-control study included the data from 2,627 infertile women with successful pregnancy and 2,627 infertile women without successful pregnancy using datasets from the Longitudinal Health Insurance Database 2000 of the National Health Insurance Research Database during 2000–2010. The odds ratios (ORs) and 95% confidence intervals (CIs) for the relationship between TCM use and successful pregnancy in infertility women were estimated using logistic regression.

**Results:**

Patients who received TCM treatment significantly increased in successful pregnancy (OR = 1.48; 95% CI = 1.31–1.66), compared with patients without TCM. Si-Wu-Tang (OR = 4.25; 95% CI = 2.18, 8.30), Gui-Zhi-Fu-Ling-Wan (OR = 3.27; 95% CI = 2.13, 5.02), and Jia-Wei-Xiao-Yao-San (OR = 3.17; 95% CI = 2.35, 4.28) were the TCM agents that were most strongly associated with successful pregnancy among infertile women.

**Conclusions:**

Our study findings indicate that TCM is associated with higher likelihood of successful pregnancy in infertile women, which is worthy of further investigation by randomized control trial.

## 1. Introduction

Infertility is an integral part of reproductive health and a priority global health issue. This condition is also a critical problem that has to be solved by many countries with low birth rates. In 2010, approximately 48.5 million infertile couples worldwide cannot have a child after five years of trying to procreate, with the highest prevalence of infertility in South Asia, North Africa/Middle East, Sub-Saharan Africa, Central Asia, and Central/Eastern Europe [1]. Procreation is one of the natural needs of human beings, and infertility is one of the important factors associated with the quality of life for married couples. Married couples may experience emotional problems because of pregnancy failure and may face the risk of social dysfunction, anxiety, and depression [2].

Taiwan has a very low birth rate, resulting from the current aging society. Taiwan is one of the countries with the lowest fertility rates globally. One cause of low birth rate is the increasing prevalence of infertility since 1990. The female total fertility rate fell from 7.04 in 1951 to 1.13 in 2017 in Taiwan [3]. Female infertility is defined as inability of a woman within childbearing age to conceive despite having frequent, unprotected intercourse for at least 1 year [4]. The inability to have children can result in psychological distress and emotional problem. Seeking alternative treatments for infertility is one solution to this public health problem.

Many causes of female infertility are menstrual abnormalities, endometriosis, pelvic adhesion, ovulatory disorders, tubal blockage, hyperprolactinemia, and uterine abnormalities [5, 6]. Western medicine therapies for female infertility include clomiphene citrate, gonadotropin-releasing hormone analogs, follicle-stimulating hormone, metformin, and aromatase inhibitor [7, 8]. Some fertility drugs have no significant effect in fertile women aged 40 years or older, while some drugs increase the risk of cancer. Traditional Chinese medicine (TCM) is commonly used for complementary or alternative therapies in these cases in Asian countries.

In addition to biomedicine, TCM is the most common form of medicine used in Taiwan. Two meta-analysis pooling randomized controlled trials (RCTs) have reported that clomiphene citrate combined with TCM significantly increased the pregnancy and ovulation rates, increased the cervical mucus score, and reduced the miscarriage rate compared with clomiphene citrate alone [9, 10]. A cross-sectional study of 8,766 new onset cases of female infertility reported the prevalence and associated factors of TCM use, as well as the top 10 Chinese herbal product (CHP) formulas for female infertility [11]. However, these studies did not explore the specific effect of TCM treatment on infertile outcomes. Thus, the present study was aimed to examine the association between TCM treatment with successful pregnancy among infertile women using population-based case-control study with matching approach to reduce the confounding effects through assembling a sample in which potential confounding factors are balanced between TCM and non-TCM groups.

## 2. Methods

### 2.1. Data Sources

The Taiwan National Health Insurance (NHI) program is a compulsory and nationwide single-payer insurance program that began in 1995. It covers almost the entire population (99.6%) of Taiwan. The Bureau of NHI has an expert review of random samples of every 50–100 outpatient and inpatient claims from each hospital and clinic in Taiwan every three months, and false reports of diagnoses have severe penalty [12]. The National Health Insurance Research Database (NHIRD) contains the following information: demographic data; dates of clinical visits; details of prescriptions and expenditure amounts of beneficiaries; diagnostic codes by the International Classification of Diseases, Ninth Revision, Clinical Modification (ICD-9-CM). Data also included detailed diagnoses and treatments provided by TCM physicians.

This study used the Longitudinal Health Insurance Database 2000 (LHID2000) of NHIRD. The LHID2000 of NHIRD sample and the entire population of NHIRD are similar in terms of age and sex distributions. The LHID2000 of NHIRD contains all outpatient and inpatient claim data of one million beneficiaries who were randomly sampled for 23 million enrollees in the NHI.

The case-control study was conducted using the registration and claim datasets for 2000–2011 from the LHID2000 of NHIRD. The female infertility in the dataset was that covered by the NHI Program. The NHI program has a committee that reviews new treatments (containing biomedicine and TCM), drugs, and procedure, and those that have evidence for their efficacy are covered. The present study was approved by the Institutional Review Board of the Public Health, Social, and Behavioral Science Committee Research Ethics Committee, China Medical University and Hospital (CMU-REC-101-012). Informed consent of the study participants was not required because the dataset used in this study consists of deidentified secondary data released for research purposes.

### 2.2. Study Subjects

A population-based case-control study was conducted using registration and claim datasets of the NHIRD for 2000–2011. The population-based case-control study can minimize the selection bias because cases are generally representative of the population of women with successful pregnancy and the controls are a representative sample, too. Infertile women were considered if a diagnostic code (ICD-9-CM code: 628) was found during 2000–2010 of the LHID2000 of NHIRD with at least three ambulatory claims. In the clinical practice of Taiwan, the diagnosis of infertility involves the evaluation of a couple. Diagnosis of infertility for a women is as inability of a couple to conceive after 12 months of regular, unprotected intercourse in women less than 35 years of age and after six months of regular intercourse without use of contraception in women 35 years and older or who have one of the following in their medical history or physical examination: history of irregular menstrual cycles over 35 days apart or no periods at all and known or suspected problems with the uterus, tubes, or other problems in the abdominal cavity such as endometriosis or adhesions [13]. For an evaluation, a couple must attend clinic simultaneously but take medical history and physical examination separately to detect the most common causes of infertility, including menstrual history, assessment of luteinizing hormone surge in urine before ovulation, and/or luteal phase progesterone level to assess ovulatory function. Then, the assessment of tubal patency and the uterine cavity is performed through hysterosalpingogram or sonohysterogram with a test of tubal patency such as hysterosalpingocontrast-sonography to detect abnormalities in the uterine cavity. This test also assesses whether the fluid passes out of the uterus and spills out of the fallopian tubes and sometimes the test itself can improve fertility for some women by flushing out and opening the fallopian tubes. If abnormal tests are found, further evaluation is needed. The other common assessments included ovarian reserve with day 3 serum follicle-stimulating hormone for determination of the quality and quantity of eggs available for ovulation, estradiol levels, anti-Müllerian hormone, antral follicle count, and/or thyroid-stimulating hormone that control reproductive processes. Some imaging tests may be needed such as pelvic ultrasound, looking for uterine or fallopian tube disease, and sonohysterogram, seeing details inside the uterus that cannot be seen on a regular ultrasound. For some rare cases, other imaging tests may be needed, including a hysteroscopy to look for uterine or fallopian tube disease, a laparoscopy to examine the fallopian tubes, ovaries and uterus, and a genetic testing to look for a genetic defect.

**Figure 1:**
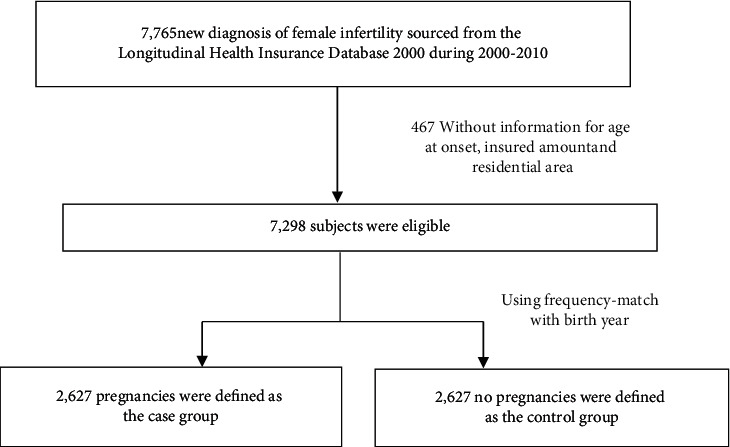
Flowchart of recruitment procedures for the current study.

A total of 7,765 infertile women were identified. After excluding 467 women without information of age at onset, insured amount, and residential area, 7,298 women were found to be eligible. Among these eligible women, 2,627 were found to have successful pregnancies and were included as the case group. After frequency-matching with birth year, 2,627 women without pregnancy were included as the control group (Figure 1).

To calculate the exposure period for TCM use, we used the following algorithm. For women with successful pregnancy, the assumed fertility date (day zero) was defined as follows: delivery date minus 270 days if no preterm birth was identified using ICD-9-CM code; delivery date minus 245 days if a preterm birth of unspecified gestational age was identified using ICD-9-CM code; and delivery date minus the upper boundary of the gestational age range in case of an indication for preterm birth by ICD-9-CM code with a specified range. For example, for an ICD-9-CM code 765.26, i.e., 31 to 32 weeks of gestation, the date of delivery minus 224 days for deliveries. The exposure period for TCM use was then defined as one year before the assumed fertility date. For those without successful pregnancy, their assumed fertility dates were assigned using their matched cases. In addition, the treatments for Western medicine with the same exposure period for TCM use were considered, including clomiphene citrate/tamoxifen, gonadotropin-releasing hormone (GnRH) agonist/GnRH antagonist, progesterone, and bromocriptine/cabergoline. To protect the patients' privacy, the NHI cryptographically scrambled data on their identities and institutions in the NHIRD.

### 2.3. Statistical Analysis

Continuous variables were reported as mean and standard deviation, while categorical variables were reported as number and percentage. The chi-square and *t*-test were used to compare differences in the baseline differences between two groups for successful pregnancy status. A univariate logistic analysis was then conducted to examine the magnitude of the associations between female infertility and TCM use as well as comorbidities. Adjusted odds ratios (ORs) and their 95% confidence intervals (CIs) were then estimated by multivariate logistic regression analysis. Stratified analyses were performed according to common gynecologic disorders (including polycystic ovary syndrome, endometriosis, irregular menstrual cycle, uterine fibroids, and dysmenorrhea), and interactions between TCM use and these gynecologic disorders were examined by their product term. In addition, we performed a sensitivity analysis to rule out the potential confounding effect of Western medicine use by excluding women with Western fertility drug use. The statistical significance was set to two-sided *p* < 0.05. All analyses were performed using SAS version 9.4 (SAS Institute., Cary, NC, USA).

## 3. Results

Baseline sociodemographic factors and comorbidities were presented according to the pregnancy status (women with pregnancy as cases and without pregnancy as controls) in Table 1. As age increased, the likelihood of successful pregnancy decreased (for 30–39 years: OR = 0.68, 95% CI = 0.60–0.76; and for ≥40 years: OR = 0.22, 0.13–0.37) (Table 2). Compared with those without TCM use during one year before assumed fertility date, TCM users were significantly associated with successful pregnancy (OR = 1.50, 1.35–1.68) without considering the other covariates. After adjustment, the results remain similar (OR = 1.48, 1.31–1.66). If we excluded women with any Western medicine use to rule out the confounding effect of Western medicine use, the multivariate-adjusted OR for TCM use was 1.60 (1.39, 1.83), which is similar to the adjusted OR for the entire sample (S2 Table). After further considering the frequency of outpatient visits, TCM users with 1–3 and >3 outpatient visits were associated with 22% and 73% increased odds of successful pregnancy, respectively, after multivariate adjustment (OR = 1.22, 1.05–1.41 and OR = 1.73, 1.50–1.98). Considering the number of days for drug prescription, TCM users with 1–14 and >14 days for drug prescription were associated with 25% and 73% increased odds of successful pregnancy, respectively, after multivariate adjustment (OR = 1.25, 1.07–1.47 and OR = 1.73, 1.51–1.97). The other significant factors after adjustment were insured amount (OR = 1.33, 1.10–1.62 for 40,000–59,9999 NT$/month; OR = 1.76, 1.35–2.30 for ≥60,000 NT$/month); insured unit (OR = 0.77, 0.63–0.94 for private enterprise employees; OR = 0.75, 0.58–0.98 for member of occupational sector; OR = 0.69, 0.49–0.98 for farmers and fishermen; OR = 0.59, 0.44–0.78 for low-income households, veterans, and other regional), comorbidity of diabetes (OR = 043, 0.22–0.84) and dysmenorrhea (OR = 0.75, 0.61–0.93), and fertility drugs of clomiphene citrate/tamoxifen (OR = 3.53, 3.06–4.08), progesterone (OR = 2.53, 1.89–3.38), and bromocriptine/cabergoline (OR = 2.20, 1.48–3.27). The distribution of combination for Western medicine and TCM used and the ORs of successful pregnancy are presented in S1 Table. Compared with those without both TCM and Western medicine use during one year before assumed fertility date, Western medicine use only, TCM use only, and both Western medicine and TCM use were significantly associated with successful pregnancy (OR: 5.20, 4.27–6.35; 1.61, 1.41–1.84; and 5.89, 4.91–7.07, respectively) without considering the other covariates. After adjustment, the results remain similar (OR = 4.96, 4.05–6.07; 1.60, 1.40–1.83; and 5.75, 4.77–6.93, respectively). Both TCM and Western medicine use significantly increase the odds of successful pregnancy over the use of just Western medicine alone (p for interaction of TCM use and Western medicine use = 0.02).

Table 3 shows that Jia-Wei-Xiao-Yao-San was the most commonly prescribed herbal formula (*n* = 253, 4.82%), followed by Wen-Jing-Tang (*n* = 213, 4.05%), Dang-Gui-Sha-Yao-San (*n* = 207, 3.94%), Zou-Gui-Wan (*n* = 152, 2.89%), Gui-Zhi-Fu-Ling-Wan (*n* = 127, 2.42%), and You-Gui-Wan (*n* = 115, 2.19%).  Table 4 shows that the effects of the top 10 TCM formulas were all significant. Jia-Wei-Xiao-Yao-San, Dang-Gui-Sha-Yao-San, Gui-Zhi-Fu-Ling-Wan, and Si-Wu-Tang had significant adjusted OR greater than 3 (the corresponding ORs and 95% CIs were as follows: 3.17, 2.35–4.28; 3.14, 2.26–4.37; 3.27, 2.13–5.02; and 4.25, 2.18–8.30), and Wen-Jing-Tang, Zou-Gui-Wan, You-Gui-Wan, Shao-Fu-Zhu-Yu-Tang, and Liu-Wei-Dihuang-Wan had significant adjusted OR greater than 2 (the corresponding ORs and 95% CIs were as follows: 2.83, 2.06–3.90; 2.47, 1.71–3.56; 2.12, 1.41–3.18; 2.54, 1.67–3.86; and 2.52, 1.51–4.21). And fertility drugs of clomiphene citrate/tamoxifen, progesterone, and bromocriptine/cabergoline remained significant (OR = 3.76, 3.26–4.34; 3.22, 2.44–4.26; 2.94, 2.00–4.32, respectively). To rule out the confounding effects of Western medicine use by excluding infertility women with any Western medicine use, the values of ORs for individual commonly used fertility drugs became larger and remain statistically significant after multivariate adjustment (S3 Table).

**Figure 2:**
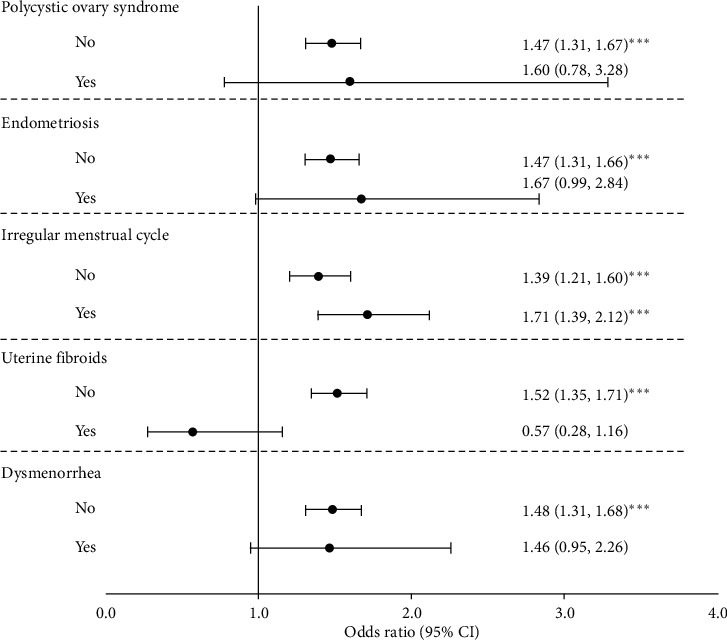
The odds ratios of pregnancy stratified by polycystic ovary syndrome, endometriosis, irregular menstrual cycle, uterine fibroids, and dysmenorrhea.

Figure 2 shows that the OR and 95% CI were significant without comorbidities for the above syndrome (the ORs and 95% CIs for no polycystic ovary syndrome, endometriosis, irregular menstrual cycle, uterine fibroids, and dysmenorrhea were as follows: 1.48, 1.31–1.67; 1.47, 1.31–1.66; 1.39, 1.21–1.60; 1.52, 1.35–1.71; and 1.48, 1.31–1.68). In addition, the effect of TCM on successful pregnancy for irregular menstrual cycle (OR = 1.71, 1.39–2.12) remained significant. No significant interaction between TCM use and the above syndromes was observed.

## 4. Discussion

This large-scale case-control study of TCM use in the treatment of female infertility consisted of 2,627 infertile women with successful pregnancy and 2,627 frequency-matched infertile women without successful pregnancy in a Taiwanese population. The results demonstrated an overall 48% increase in successful pregnancy for women receiving TCM compared with those not receiving TCM. The infertile women with diabetes and endometriosis were associated with decreased likelihood for successful pregnancy. The top four prescriptions with the strongest strength of association with successful pregnancy were Si-Wu-Tang, Gui-Zhi-Fu-Ling-Wan, Jia-Wei-Xiao-Yao-San, and Dang-Gui-Sha-Yao-San with corresponding ORs of 4.25, 3.27, 3.17, and 3.14. Subgroup analysis revealed that the magnitude of association between TCM use and successful pregnancy in women with endometriosis and irregular menstrual cycle was slightly higher.

The 10 most common TCM prescriptions used by female infertility in this study were similar to those reported by Lin et al. who explored the TCM use for dysfunctional uterine bleeding [11] in 7 common prescriptions. This study was also consisted with that of Hung et al. who investigated the TCM use for female infertility with 10 common prescriptions [14]. However, the strength of association between outcomes and TCM prescription and subgroup analysis based on the status of gynecological diseases were not examined in these two studies. We found that Jia-Wei-Xio-Yao-San, Wei-Jing-Tang, Dang-Gui-Sha-Yao-San, and Gui-Zhi-Fu-Ling-Wan exhibited the strongest effect on improving female fertility. Jia-Wei-Xio-Yao-San was also reported to be the most common prescription used in treating patients with colon or breast cancer [15–17] and dysfunctional uterine bleeding [11].

This study is the first large-scale case-control to report the association between successful pregnancy and TCM used in infertile women in Taiwanese society. Our sample is representative of the general population. Given the high coverage rate of NHI on TCM healthcare, the TCM prescription data are completely recorded in the NHI databases. Thus, the likelihood of potential recall or differential measurement bias was minimized. The TCM prescription includes herbal or formulas prescribed by board-certified TCM physicians. The complete information on TCM prescription facilitates the investigation of the independent effects of each type of prescription on the pregnancy outcome in female infertility. This process is more comprehensive than merely evaluating a particular type of TCM prescription.

Our study reports the top 10 commonly prescribed formulas for female infertility. A previous epidemiological prospective cohort study noted that Jia-Wei-Xio-Yao-San ameliorated depression in menopausal women compared with those administered antidepressants by increasing the serum TNF-*α* after 12 weeks of treatment [18]. In another multicenter, randomized, double-blinded, placebo-controlled study, persons treated with multicompound extract of Jia-Wei-Xio-Yao-San showed significantly decreased anxiety symptoms compared with those with individual extract mixture, and placebo group [19].

The findings indicated that Si-Wu-Tang was the TCM formula exerted strongest association with successful pregnancy. Si-Wu-Tang was administered for blood deficiency, which is particularly common in women because of the loss of menstrual blood. Some of the ingredients of Si-Wu-Tang include Radix Rehmanniae Preparata, Radix Paeoniae Lactiflorae, and Radix *Angelicae sinensis*. Radix Rehmanniae, which is an important ingredient of diverse TCM formulas, has anti-inflammation and antioxidation effect to alleviate dermatitis [20], autoimmune disease [5], and allergic diseases [6]. Moreover, this compound has an effect on the activation of the parasympathetic nervous system determined by heart rate variability [19].

The goal of the subgroup analysis is to assess whether there exists consistency of or large differences in the magnitude of treatment effect among different subgroups of patients. It provides more precise findings when the health professionals applied these results to patients in clinical practice. The results of our subgroup analysis found that the association between TCM use and successful pregnancy is only consistent across categories of regular and irregular menstrual cycles. The association between TCM and successful pregnancy was not significant in women with polycystic ovary syndrome, endometriosis, or dysmenorrhea even though the magnitude of OR was slightly larger than or similar to those in women without these corresponding comorbidities. These results may be due to small sample size for women with polycystic ovary syndrome, endometriosis, or dysmenorrhea. It seems that uterine fibroids exert antagonistic interaction on TCM use and successful pregnancy although this interaction is not significant. To provide this association in infertile women with these comorbidities, a larger scale study is needed. Given the results of the subgroup analysis, our study's findings may not be generalized to infertile women with polycystic ovary syndrome, endometriosis, uterine fibroids, or dysmenorrhea.

An animal study first demonstrated that Dang-Gui-Sha-Yao-San improved depression-like behavior, such as increased open-field activities, shortened immobility time, and percentage of sugar preference compared with the stress control group by the potential mechanisms of decreasing central arginine vasopressin and the expression of AVP mRNA [21]. In an in vitro study using rat uterine smooth muscle, Dang-Gui-Sha-Yao-San also had its antagonistic effect on contraction caused by KCL depolarization dysmenorrhea by suppressing smooth muscle contractions of uterine [22]. In addition, Dang-Gui-Sha-Yao-San had been shown in vivo that women with luteal insufficiency, which was determined by the daily measurement of basal body temperature and plasma progesterone levels, had improved their insufficiency for luteal phase [23]. In an observational study of 39 consecutive patients with climacteric disorders diagnosed by the Kupperman index, the administration of 12-week Gui-Zhi-Fu-Ling-Wan relieved symptoms of climacteric disorders, such as vasomotor symptoms induced by estrogen-like activity [24]. Moreover, the therapeutic effect of molecular mechanisms for Gui-Zhi-Fu-Ling-Wan had been explored in a mouse study that compared in vivo and in vitro models. This study demonstrated that Gui-Zhi-Fu-Ling-Wan exerted its effect on decreasing spontaneous uterine contraction induced by oxytocin PGF22*α* in a dose-response relationship for attenuating dysmenorrhea [25].

Several limitations should be considered. First, our study was not a randomization study but an observational one. Thus, this work lacks randomization. A potential bias caused by unknown or residual confounding variables of age at diagnosis and TCM use status one year before diagnosis is possible. In this study, we used multivariate analysis to control for the potential residual confounding effects of matching approach. Second, the data source is from the clinical practice of administrative claim data, and potential measurement errors may exist in defining baseline comorbidity, because disease ascertainment was based on ICD-9 codes. Only three ICD-9 codes can be recorded in ambulatory care. Therefore, the prevalence of some baseline comorbidity may be underestimated. However, the bias arising from the underestimation of comorbidity is less likely, because the likelihood of underestimation of comorbidity associated with TCM use is very likely to be random. Third, our study did not have laboratory data and chart records, such as imaging data and physician notes. Thus, we cannot determine the cohorts' hormone level. Finally, nondifferential misclassification of TCM users may occur, because the over-the-counter (OTC) TCM use was not covered by the NHI program. Thus, prevalence of TCM utilization could be underestimated if infertile women looked for OTC TCM use because infertile women who used OTC TCM were not identified in the NHI program. However, OTC TCM use is common for health promotion and common cold, but not for fertility problem. The magnitude of underestimation may be small. Moreover, a small proportion of outpatient visits at some noncontracted clinics were not considered. The measurement error problem is small, because only less than 10% of TCM clinics are not contracted with NHI. Patients may have health food containing herbs available in our dataset. Under the NHI program, only products of Chinese herbal certified by good manufacturing practice standards are considered. Certified products of Chinese herbal had standardized constituents and dosages, which enhanced the standardization measurements of TCM formula. Given the above conditions, some women who used TCM that was not covered by NHI program may be misclassified as non-TCM users. Hence, the present study may cause a misclassification error of TCM use status. If TCM use did enhance the successful pregnancy likelihood, this misclassification error would result in underestimating the effect of TCM use, which would be a less threat to the validity of our study's findings because the true association of TCM use and successful pregnancy would be stronger.

## 5. Conclusions

We demonstrated TCM as an adjunctive therapy is common for infertile women. The results suggested that TCM was associated with higher likelihood of the successful pregnancy in infertile women, which is worthy of further investigation by randomized control trial. Our study suggests that TCM may be used in clinical management for treating female infertility.

## Figures and Tables

**Table 1:** Infertility patient characteristics with or without pregnancy.

Characteristic	Infertility women (*n* = 5,254)		*p* value
	No successful pregnancy (*n* = 2,627)	Successful pregnancy (*n* = 2,627)	
*Birth year*			1.00
1959–1969	518 (19.72)	518 (19.72)	
1970–1979	1699 (64.67)	1699 (64.67)	
1980–1988	410 (15.61)	410 (15.61)	

*Age at diagnosis*			<0.001
20–29	1166 (44.39)	1427 (54.32)	
30–39	1376 (52.38)	1181 (44.96)	
≥40	85 (3.24)	19 (0.72)	

*One-year period before assumed fertility date*			<0.001
TCM nonusers	1348 (51.31)	1083 (41.23)	
TCM users	1279 (48.69)	1544 (58.77)	
Number of outpatient visits✝	6.64 ± 7.78	7.62 ± 8.24	
Days of administration✝	36.08 ± 47.05	46.47 ± 56.34	

*Insured amount (NT$/month)*			<0.001
<20,000	1066 (40.58)	935 (35.59)	
20,000–39,999	1032 (39.28)	1008 (38.37)	
40,000–59,999	388 (14.77)	488 (18.58)	
≥60,000	141 (5.37)	196 (7.46)	

*Urban level*			0.09
1	910 (34.64)	946 (36.01)	
2	861 (32.78)	796 (30.3)	
3	409 (15.57)	458 (17.43)	
≥4	447 (17.02)	427 (16.25)	

*Residential area*			0.88
Northern	401 (15.26)	399 (15.19)	
Taipei	995 (37.88)	1032 (39.28)	
Central	459 (17.47)	436 (16.6)	
Southern	333 (12.68)	337 (12.83)	
Eastern	39 (1.48)	41 (1.56)	
Kao-Ping	400 (15.23)	382 (14.54)	

*Insured unit*			<0.001
Government, school employees	248 (9.44)	357 (13.59)	
Private enterprise employees	1597 (60.79)	1661 (63.23)	
Member of occupational	324 (12.33)	270 (10.28)	
Farmers, fishermen	139 (5.29)	114 (4.34)	
Low-income households veterans, other regionals	319 (12.14)	225 (8.56)	

*Comorbidities*			
Hypertension	27 (1.03)	13 (0.49)	0.04
Diabetes	40 (1.52)	14 (0.53)	<0.001
Hypercholesterolemia	32 (1.22)	19 (0.72)	0.09
Obesity	20 (0.76)	10 (0.38)	0.10
Cerebral vascular disease	8 (0.30)	6 (0.23)	0.79
COPD	143 (5.44)	138 (5.25)	0.81
Renal failure	6 (0.23)	3 (0.11)	0.50
Tobacco use	2 (0.08)	1 (0.04)	1.00
Anemia	22 (0.84)	17 (0.65)	0.52
Polycystic ovary syndrome	104 (3.96)	104 (3.96)	1.00
Endometriosis	181 (6.89)	133 (5.06)	0.006
Irregular menstrual cycle	883 (33.61)	862 (32.81)	0.56
Uterine fibroids	127 (4.83)	84 (3.20)	0.003
Dysmenorrhea	253 (9.63)	203 (7.73)	0.01

*Fertility drugs*			
Clomiphene citrate/tamoxifen	340 (12.94)	959 (36.51)	<0.001
GnRH agonist/GnRH antagonist	0 (0.00)	2 (100.00)	0.50
Progesterone	70 (2.66)	220 (8.37)	<0.001
Bromocriptine/cabergoline	3 (1.41)	110 (4.19)	<0.001

TCM: traditional Chinese medicine; CAD: coronary artery diseases; COPD: chronic obstructive pulmonary disease. *p* values for chi-square or Fisher's exact test; ✝: mean ± standard deviation for TCM users.

**Table 2:** Unadjusted and adjusted odd ratios and 95% confidence interval of successful pregnancy among women with infertility.

Characteristic	Unadjusted OR (95% CI)	Adjusted OR (95% CI)
*Age at diagnosis*		
20–29	1.00	1.00
30–39	0.70 (0.63, 0.78)^*∗∗∗*^	0.68 (0.60, 0.76)^*∗∗∗*^
≥40	0.18 (0.11, 0.30)^*∗∗∗*^	0.22 (0.13, 0.37)^*∗∗∗*^

*One-year period before assumed fertility date*		
TCM nonusers	1.00	1.00
TCM users	1.50 (1.35, 1.68)^*∗∗∗*^	1.48 (1.31, 1.66)^*∗∗∗*^

*Number of outpatient visits*		
0	1.00	1.00✝
1–3	1.25 (1.09, 1.43)^*∗∗*^	1.22 (1.05, 1.41)^*∗∗*^
>3	1.74 (1.53, 1.98)^*∗∗∗*^	1.73 (1.50, 1.98)^*∗∗∗*^

*Number of days for drug prescription*		
0	1.00	1.00✝
1–14	1.30 (1.12, 1.51)^*∗∗∗*^	1.25 (1.07, 1.47)^*∗∗∗*^
>14	1.76 (1.55, 1.99)^*∗∗∗*^	1.73 (1.51, 1.97)^*∗∗∗*^

*Insured amount (NT$/month)*		
<20,000	1.00	1.00
20,000–39,999	1.11 (0.98, 1.26)	1.06 (0.92, 1.23)
40,000–59,999	1.43 (1.22, 1.68)^*∗∗∗*^	1.33 (1.10, 1.62)^*∗∗*^
≥60,000	1.59 (1.26, 2.00)^*∗∗∗*^	1.76 (1.35, 2.30)^*∗∗∗*^

*Urban level*		
1	1.00	1.00
2	0.89 (0.78, 1.02)	0.87 (0.74, 1.02)
3	1.08 (0.92, 1.27)	1.09 (0.90, 1.33)
≥4	0.92 (0.78, 1.08)	0.98 (0.79, 1.21)

*Residential area*		
Taipei	1.00	1.00
Northern	0.96 (0.81, 1.13)	1.02 (0.84, 1.24)
Central	0.92 (0.78, 1.07)	0.88 (0.72, 1.07)
Southern	0.98 (0.82, 1.16)	1.06 (0.86, 1.32)
Eastern	1.01 (0.65, 1.59)	0.81 (0.49, 1.34)
Kao-Ping	0.92 (0.78, 1.09)	0.99 (0.82, 1.20)

*Insured unit*		
Government, school employees	1.00	1.00
Private enterprise employees	0.72 (0.61, 0.86)^*∗∗∗*^	0.77 (0.63, 0.94)^*∗*^
Member of occupational	0.58 (0.46, 0.73)^*∗∗∗*^	0.75 (0.58, 0.98)^*∗*^
Farmers, fishermen	0.57 (0.42, 0.77)^*∗∗∗*^	0.69 (0.49, 0.98)^*∗*^
Low-income households veterans, other regionals	0.49 (0.39, 0.62)^*∗∗∗*^	0.59 (0.44, 0.78)^*∗∗*^

*Comorbidities*		
Hypertension	0.48 (0.25, 0.93)^*∗*^	0.84 (0.41, 1.73)
Diabetes	0.35 (0.19, 0.64)^*∗∗∗*^	0.43 (0.22, 0.84)^*∗*^
Hypercholesterolemia	0.59 (0.33, 1.05)	0.90 (0.48, 1.70)
Obesity	0.50 (0.23, 1.07)	0.71 (0.31, 1.59)
Cerebral vascular disease	0.75 (0.26, 2.17)	0.61 (0.19, 1.96)
COPD	0.96 (0.76, 1.23)	0.93 (0.71, 1.20)
Renal failure	0.50 (0.13, 2.00)	0.61 (0.13, 2.81)
Tobacco use	0.50 (0.05, 5.52)	0.65 (0.06, 7.46)
Anemia	0.77 (0.41, 1.46)	0.70 (0.36, 1.38)
Polycystic ovary syndrome	1.00 (0.76, 1.32)	0.97 (0.72, 1.32)
Endometriosis	0.72 (0.57, 0.91)^*∗∗*^	0.88 (0.68, 1.14)
Irregular menstrual cycle	0.97 (0.86, 1.08)	0.91 (0.81, 1.04)
Uterine fibroids	0.65 (0.49, 0.86)^*∗∗*^	0.86 (0.63, 1.17)
Dysmenorrhea	0.79 (0.65, 0.95)^*∗*^	0.75 (0.61, 0.93)^*∗∗*^

*Fertility drugs*		
Clomiphene citrate/tamoxifen	3.87 (3.37, 4.44)^*∗∗∗*^	3.53 (3.06, 4.08)^*∗∗∗*^
GnRH agonist/GnRH antagonist	—	—
Progesterone	3.34 (2.54, 4.39)^*∗∗∗*^	2.53 (1.89, 3.38)^*∗∗∗*^
Bromocriptine/cabergoline	3.06 (2.10, 4.46)^*∗∗∗*^	2.20 (1.48, 3.27)^*∗∗∗*^

OR: odd ratio; ^*∗*^*p* < 0.05; ^*∗∗*^*p* < 0.01; ^*∗∗∗*^*p* < 0.001. ✝: multivariate-adjusted for age at diagnosis, insured amount, urban level, residential area, insured unit, and comorbidities.

**Table 3:** The TCM name, ingredients or generic name, and functional classification of the commonly used TCM prescriptions in patients with female infertility.

TCM name	Ingredients or generic name	Functional classification	No. of users (%#)	
			Successful pregnancy	No successful pregnancy
Jia-Wei-Xiao-Yao-San	Moutan Radicis Cortex; Radix Paeoniae Rubra; Bupleuri Radix; *Angelicae sinensis* Radix; *Atractylodis ovatae* Rhizoma; Poria; Glycyrrhizae Radix; Zingiberis Rhizoma Recens; Menthae Herba	Shugan Jieyu, heat-clearing and nourishing blood	194 (7.38%)	59 (2.25%)

Wen-Jing-Tang	Cinnamomi Ramulus; Evodiae Fructus; Ligustici Rhizoma; *Angelicae sinensis* Radix; Paeoniae Radix; Zingiberis Rhizoma Recens; Moutan Radicis Cortex; Ophiopogonis Tuber; Pinelliae Tuber; Ginseng Radix; Glycyrrhizae Radix; Asini Corii Gelatinum	Promoting blood circulation and removing blood stasis, warming meridian and removing cold, benefiting qi, and nourishing blood	160 (6.09%)	53 (2.02%)

Dang-Gui-Sha-Yao-San	*Angelicae sinensis* Radix; Paeoniae Radix; Poriz; *Atractylodis ovatae* Rhizoma; Alismatis Rhizoma; Ligustici Rhizoma	Nourishing blood and regulating the liver, and tonifying the spleen and dampness	158 (6.01%)	49 (1.87%)

Zou-Gui-Wan	Rhizoma Rehmanniae Preparata; Rhizoma Dioscoreae; Fructus Lycii; Fructus Corni; Radix Cyathulae; Semen Cuscutae; Colla Cornus Cervi; Colla Plastri Testudinis	Nourishing yin and tonifying the kidney, and filling essence	110 (4.19%)	42 (1.60%)

Gui-Zhi-Fu-Ling-Wan	Cinnamomi Ramulus; Poria; Moutan Radicis; Persicae Semem; Paeoniae Radix Rubra	Promoting blood circulation and removing blood stasis removing blocked mass	99 (3.77%)	28 (1.07%)

You-Gui-Wan	Rhizoma Rehmanniae Preparata; Rhizoma Dioscoreae; Fructus Lycii; Semen Cuscutae; Colla Cornus Cervi; Fructus Corni; *Angelicae sinensis* Radix; Radix Aconiti Preparata; *Cinnamomum cassia* Blume; *Eucommia ulmoides* Oliv.	Warming kidney yang, replenishing essence, and enriching the blood	80 (3.05%)	35 (1.33%)

Shao-Fu-Zhu-Yu-Tang	Fructus Foenicuii; Rhizoma Zingiberis; Rhizoma Corydalis; *Angelicae sinensis* Radix; Rhizoma Ligustici Chuanxiong; Cortex Cinnamomi; Radix Paeoniae Rubra; Pollen Typhae; Faeces Trogopterorum	Promoting blood circulation and removing blood stasis, warming meridian, and pain relief	83 (3.16%)	32 (1.22%)

Liu-Wei-Dihuang-wan	Radix Rehmanniae Preparata; Corni Fructus.; Dioscoreae Rhizoma; Alismatis Rhizoma; Poria; Moutan Cortex.	Nourishing the liver and kidney	57 (2.17%)	21 (0.80%)

Gui-Pi-Tang	*Atractylodis ovatae* Rhizoma; Poria; Astragali Radix; Ginseng Radix; Glycyrrhizae Radix; Saussureae Radix; *Angelicae sinensis* Radix; Polygalae Radix; Longanae Arillus; Zizyphi Spinosi Semen	Replenishing qi and blood, tonifying the spleen, and nourishing the heart	49 (1.87%)	25 (0.95%)

Si-Wu-Tang	*Angelicae sinensis* Radix; Rehmanniae Radix; Paeoniae Radix; Ligustici Rhizoma	Replenishing blood and regulating menstruation	46 (1.75%)	11 (0.42%)

#: number of users divided by the number of successful pregnancy/unsuccessful pregnancy.

**Table 4:** Unadjusted and adjusted odd ratios of successful pregnancy for individual commonly used fertility drugs.

Fertility drugs	*N*	%	Unadjusted OR (95% CI)	Adjusted OR (95% CI)
*TCM prescription*				
Jia-Wei-Xiao-Yao-San	253	4.82	3.47 (2.58, 4.67)^*∗∗∗*^	3.17 (2.35, 4.28)^*∗∗∗*^
Wen-Jing-Tang	213	4.05	3.15 (2.30, 4.32)^*∗∗∗*^	2.83 (2.06, 3.90)^*∗∗∗*^
Dang-Gui-Sha-Yao-San	207	3.94	3.37 (2.43, 4.66)^*∗∗∗*^	3.14 (2.26, 4.37)^*∗∗∗*^
Zou-Gui-Wan	152	2.89	2.69 (1.88, 3.85)^*∗∗∗*^	2.47 (1.71, 3.56)^*∗∗∗*^
Gui-Zhi-Fu-Ling-Wan	127	2.42	3.64 (2.38, 5.55)^*∗∗∗*^	3.27 (2.13, 5.02)^*∗∗∗*^
You-Gui-Wan	115	2.19	2.33 (1.56, 3.47)^*∗∗∗*^	2.12 (1.41, 3.18)^*∗∗∗*^
Shao-Fu-Zhu-Yu-Tang	115	2.19	2.64 (1.75, 3.99)^*∗∗∗*^	2.54 (1.67, 3.86)^*∗∗∗*^
Liu-Wei-Dihuang-Wan	78	1.48	2.75 (1.66, 4.55)^*∗∗∗*^	2.52 (1.51, 4.21)^*∗∗∗*^
Gui-Pi-Tang	74	1.41	1.98 (1.22, 3.21)^*∗∗*^	1.69 (1.04, 2.77)^*∗*^
Si-Wu-Tang	57	1.08	4.24 (2.19, 8.20)^*∗∗∗*^	4.25 (2.18, 8.30)^*∗∗*^

*Western medicine*				
Clomiphene citrate/tamoxifen	1299	24.72	3.87 (3.37, 4.44)^*∗∗∗*^	3.76 (3.26, 4.34)^*∗∗∗*^
GnRH agonist/GnRH antagonist	2	0.04	—	—
Progesterone	290	5.52	3.34 (2.54, 4.39)^*∗∗∗*^	3.22 (2.44, 4.26)^*∗∗∗*^
Bromocriptine/cabergoline	147	2.80	3.06 (2.10, 4.46)^*∗∗∗*^	2.94 (2.00, 4.32)^*∗∗∗*^

OR: odd ratio; 95% CI: 95% confidence interval; TCM: traditional Chinese medicine; ^*∗*^*p* < 0.05; ^*∗∗*^*p* < 0.01; ^*∗∗∗*^*p* < 0.001. #: number of users divided by the total sample size. Multivariate-adjusted for age at diagnosis, insured amount, urban level, residential area, insured unit, and comorbidities.

## Data Availability

The data used to support the findings of this study are included within the article.

## Abbreviations

TCM: Traditional Chinese medicine
CHP: Chinese herbal product
NHI: National Health Insurance
NHIRD: National Health Insurance Research Database
ICD-9-CM: International Classification of Diseases, Ninth Revision, Clinical Modification
LHID2000: Longitudinal Health Insurance Database 2000
GnRH: Gonadotropin-releasing hormone
ORs: Odds ratios
CIs: Confidence intervals.

## Supplementary Materials

Supplementary Materials Supplementary Materials. Table S1: infertility women's medication use based on with or without pregnancy status. Table S2: unadjusted and adjusted odds ratios and 95% confidence intervals of successful pregnancy among infertility women for TCM use by excluding women with Western fertility drugs use (*n* = 3,731). Table S3: unadjusted and adjusted odds ratios of successful pregnancy for individual commonly used fertility drugs by excluding women with Western fertility drugs use (*n* = 3,731).
