# Supplementary material for: Traditional Chinese Medicine Treatment Associated with Female Infertility in Taiwan: A Population-Based Case-Control Study
Source: Evid Based Complement Alternat Med. 2020 Dec 8;2020:3951741. doi: 10.1155/2020/3951741 (PMC7749771; doi:10.1155/2020/3951741)
Supplement: Supplementary Materials — Table S1: infertility women's medication use based on with or without pregnancy status. Table S2: unadjusted and adjusted odds ratios and 95% confidence intervals of successful pregnancy among infertility women for TCM use by excluding women with Western fertility drugs use (n = 3,731). Table S3: unadjusted and adjusted odds ratios of successful pregnancy for individual commonly used fertility drugs by excluding women with Western fertility drugs use (n = 3,731). [file 3951741.f1.docx]

**Table S1.** Infertility women’s medication use based on with or without pregnancy status.

|  | Infertility women (n=5,254) | |  |  |  |
| --- | --- | --- | --- | --- | --- |
| Characteristic | No successful pregnancy (n=2,627) | Successful pregnancy  (n=2,627) | P value | Unadjusted OR (95% CI) | Adjusted  OR (95% CI) |
| Infertility medication use |  |  | <0.001 |  |  |
| Western medicine alone | 176 (6.70) | 475 (18.08) |  | 5.20***  (4.27, 6.35) | 4.96***  (4.05, 6.07) |
| TCM alone | 1064 (40.50) | 887 (33.76) |  | 1.61***  (1.41, 1.84) | 1.60***  (1.40, 1.83) |
| Both | 215 (8.18) | 657 (25.01) |  | 5.89***  (4.91, 7.07) | 5.75***  (4.77, 6.93) |
| Both not | 1172 (44.61) | 608 (23.14) |  | 1.00 | 1.00 |

P for the interaction of Western medicine use and TCM use=0.02.

Multivariate-adjusted for age at diagnosis, insured amount, urban level, residential area, insured unit, and comorbidities.

***: P<0.001.

**Table S2.** Unadjusted and adjusted odd ratios and 95% confidence interval of successful pregnancy among infertility women for TCM use by excluding women with Western fertility drugs use (n=3,731).

| Characteristic | Unadjusted  OR (95% CI) | Adjusted  OR (95% CI) |
| --- | --- | --- |
| One-year period before assumed fertility date | |  |
| TCM Nonusers | 1.00 | 1.00 |
| TCM Users | 1.61 (1.41, 1.83)*** | 1.60 (1.39, 1.83)*** |

Multivariate-adjusted for age at diagnosis, insured amount, urban level, residential area, insured unit, and comorbidities.

***: P<0.001.

**Table S3.** Unadjusted and adjusted odd ratios of successful pregnancy for individual commonly used fertility drugs by excluding women with Western fertility drugs use (n=3,731).

| Fertility drugs | N | % | Unadjusted OR  (95% CI) | Adjusted OR  (95% CI) |
| --- | --- | --- | --- | --- |
| ***TCM prescription*** |  |  |  |  |
| Jia-Wei-Xiao-Yao-San | 151 | 4.05 | 4.56 (3.15, 6.61)*** | 4.17 (2.87, 6.09)*** |
| Wen-Jing-Tang | 138 | 3.70 | 4.47 (3.04, 6.58)*** | 4.11 (2.78, 6.09)*** |
| Dang-Gui-Sha-Yao-San | 126 | 3.38 | 4.25 (2.85, 6.33)*** | 4.12 (2.74, 6.19)*** |
| Zou-Gui-Wan | 99 | 2.65 | 3.56 (2.31, 5.49)*** | 3.22 (2.07, 5.02)*** |
| Gui-Zhi-Fu-Ling-Wan | 74 | 1.98 | 4.46 (2.63, 7.54)*** | 3.84 (2.25, 7.54)*** |
| You-Gui-Wan | 62 | 1.66 | 2.40 (1.44, 4.02)*** | 2.17 (1.28, 3.67)** |
| Shao-Fu-Zhu-Yu-Tang | 60 | 1.61 | 3.05 (1.77, 5.23)*** | 2.80 (1.61, 4.86)*** |
| Liu-Wei-Dihuang-Wan | 45 | 1.21 | 4.70 (2.38, 9.31)*** | 4.25 (2.12, 8.55)*** |
| Gui-Pi-Tang | 47 | 1.26 | 3.24 (1.75, 6.00)*** | 2.77 (1.45, 5.20)** |
| Si-Wu-Tang | 34 | 0.91 | 7.09 (2.93, 17.17)*** | 7.48 (3.03, 18.46)*** |

OR: odd ratio; 95% CI: 95% confidence interval; TCM: traditional Chinese medicine; *: p<0.05; **: p<0.01; ***: p<0.001. #: number of users divided by the total sample size. Multivariate-adjusted for age at diagnosis, insured amount, urban level, residential area, insured unit, and comorbidities.
